# Illegal and Legal Parrot Trade Shows a Long-Term, Cross-Cultural Preference for the Most Attractive Species Increasing Their Risk of Extinction

**DOI:** 10.1371/journal.pone.0107546

**Published:** 2014-09-16

**Authors:** José L. Tella, Fernando Hiraldo

**Affiliations:** Department of Conservation Biology, Estación Biológica de Doñana (CSIC), Sevilla, Spain; University of Kent, United States of America

## Abstract

Illegal trade constitutes a major threat for a variety of wildlife. A criminology framework has been recently applied to parrot poaching in Mexico, suggesting an opportunistic crime in which the most abundant and accessible species, and not the rare or highly priced species, were poached more often. We analyzed this information, together with additional long-term data (1981–2005) on both the legal and illegal trade of the 22 Mexican parrot species (n = 31,019 individuals), using multivariate statistics and hypothesis-testing approaches. Our results showed a selective capture of parrot species attending to their attractiveness. Parrot species widely differed in attractiveness to people (as reflected by their combined measures of body size, coloration, and ability to imitate human speech), and their attractiveness strongly correlated with their prices both in the Mexican and US markets. The most attractive and valuable species (amazons and macaws) were disproportionally caught attending to the number of years they were legally trapped. Similar patterns were found for parrots poached for the domestic Mexican market, for those smuggled to the USA, and for those legally exported before or after 1992, when the USA ban led parrot exports to be mostly directed to European countries. Finally, the long-term cross-cultural preference for the most attractive species has led them to be among the most threatened species today. Since current parrot poaching mostly responds to local demand, socio-ecological work is needed to reverse the long-standing pet-keeping tradition that may decimate the most desired species in Neotropical countries.

## Introduction

Overexploitation caused by the wildlife trade is listed among the major threats for wildlife, including birds [1]. The case of parrots (Order Psittaciforms) is particularly concerning. The colorful plumage and ability to talk make them heavily sought after as pets, and thus at least 259 species of parrots have been internationally traded worldwide, involving millions of individuals in recent decades [2]. Although more detailed studies on the effects of the parrot trade on wild populations and on harvesting sustainability are lacking [3], [4], wildlife trade is thought to contribute to the fact that nearly 30% of the 355 species of parrots are currently threatened with extinction [5]. This large-scale problem has attracted the attention of conservation biologists [2]–[8] and, more recently, of conservation criminologists [9]–[13]. Although international bans [7], [14] have reduced the legal trade of birds in recent decades [8], illegal parrot poaching remains highly active in several countries [13].

Wildlife crime is defined as the taking, trading, exploiting or possessing of the world's wild flora and fauna in contravention of national and international laws [9]. Conservation criminology, recently developed as a new branch of criminology [15], [16], may help to get at the roots of the illegal wildlife trade and thus complement conservation biology approaches for the more effective prevention, persecution, and management of poaching activities [9]. Recently, Pires and Clarke [12] extended the CRAVED model in criminology to the crime of parrot poaching. CRAVED is a general model of theft choices drawn from routine activity and rational choice theory, which measures several components of stolen objects (concealable, removable, available, valuable, enjoyable, disposable, i.e. CRAVED) and has been successful in explaining the targets of a variety of thefts [12]. Within this framework, and given that parrot species differ widely in their attractiveness and rarity, Pires and Clarke [12] tested whether there are preferences for poaching particular species (indicating a targeted crime) or, alternatively, whether the most poached species are those more widely available in terms of abundance and accessibility and more easily removable in terms of ease of capture (indicating an opportunistic crime). This is not a trivial question for conservation, since a preference for rare and more valuable species may drive an anthropogenic Allee effect that accelerates extinction risk [17], as was the case in the Spix's macaw (*Cyanopsitta spixii*), now extinct in the wild [5]. By analyzing the larger and more comprehensive data set on illegal parrot trade currently available, gathered from Mexico by Cantú *et al.* [18], Pires and Clarke [12] concluded that parrot poaching is an opportunistic crime affecting the more abundant and accessible species more so than the rarer or more valuable species. However, their statistical approach (univariate correlations) did not allow for the testing of combined effects of explanatory variables on the numbers of poached parrots. Here, we reanalyze the same data set, along with additional data on both the illegal and legal trade of parrots in Mexico, using a hypothesis-testing approach and more discerning multivariate statistical tools. Our results change some previous conclusions: the most attractive species were highly valued and traded -both legally and illegally- over their relative legal availability. This preference for particular species does not support an opportunistic crime and has important conservation implications since, after decades of trade, species preferred in both Mexico and other countries are currently among the most threatened.

## Methods

### Ethics Statement

This work analyses published data sets and thus did not require specific permits.

### Trade Data

Pires and Clarke [12] used as the response variable for analyses the estimated number of parrots captured annually from the 22 species of Mexican parrots as reported by Cantú *et al.* [18]. However, as authors recognized, these poaching estimates have some important limitations: interviews with trappers and police might be biased, thus making extrapolations to the whole country questionable, and estimates were grouped within nine round-number categories, thus artificially reducing variance among species and impeding finer statistical analyses. Therefore, we instead used the number of parrots illegally captured and seized (*n* = 13,375 identified parrots) by 513 trained wildlife agents (PROFEPA) throughout the country between 1995 and 2005 (Table 9.8 in [18]; 303 parrots additionally seized by PGR were not considered because species were not identified for half of all individuals). Although the number of seized parrots per species correlated well with the estimated annual captures used by Pires and Clarke [12] (Spearman correlation, *r* = 0.74, *P*<0.001, *n* = 22), our variable allows us to perform adequate GLM models (see below) and direct comparisons with additional trade data sources.

We analyzed two additional data sources for a better understanding of potential preferences for particular parrot species. First, we used the number of parrots seized at the Mexican border upon attempts to illegally bring them into the USA (*n* = 1,600 identified parrots) between 1992 and 2005 (Table 9.12 in [18]). Second, we obtained data on the number of Mexican parrots legally exported (*n* = 16,044) from 1981 (the first year for which CITES compiled records) to 2005 (www.cites.org). We split legal international trade data into two periods: before 1992 (when the US banned the parrot trade, [7]) and from 1992 until 2005 (when Europe banned the import of wild birds, [14]), since the international demand of particular parrot species could have changed after these two major trade bans. Later years were not considered as only 312 individual Mexican parrots were exported between 2006 and 2012 (www.cites.org). A number of exported individuals were identified as *Amazona ochrocephala* before this species was split into two separate species (*A. oratrix* and *A. auropalliata*). For these individuals, we made assignments based on the known proportion of *A. oratrix* and *A. auropalliata* amongst traded individuals reported by CITES.

### Species-specific Variables

We followed the approach of Pires and Clarke [12] and thus used the same explanatory variables reported in their Table A.1, slightly modifying some as explained below. The CRAVED components and how they were measured are resumed as follows (see Pires and Clarke [12] for detailed explanations; note authors were not able to obtain a measure of "concealable"):

#### Removable

Species had been scored into four levels based on the level of difficulty in accessing their nests, given that many captures come from nest poaching [18]. However, we scored them into two groups (easy or difficult to access) to reduce the number of levels in the factor and thus avoid the statistical problem of a small number of cases for the original scores.

#### Available

As a proxy of *accessibility*, the authors measured the overlap between the distribution of each parrot species in Mexico and human populations using GIS tools. As a proxy of *abundance*, they used the number of years between 1979 and 2005 in which the Mexican authorities permitted each species to be legally trapped, assuming the trapping of more abundant species was allowed in more years [18]. This interpretation is however questionable (see Discussion).

#### Valuable and disposable

Pires and Clarke [12] found it difficult to separate these components, and thus scored species based on their price (low, high) and conservation status (non-threatened, threatened) to obtain a single binary variable, which would reflect their value to collectors in the bird trade. Following our hypotheses-based statistical approach (see below), we used these variables separately. Current conservation status was also coded in a binary manner based on the 2013 IUCN Red List (www.iucnredlist.org), taking into account that the scarlet macaw (*Ara macao*) is not globally threatened but that the Mexican subspecies is endangered [19]. Prices of parrots, both in Mexico and the US [18], were however treated as two continuous variables.

#### Enjoyable

**Figure 1:**
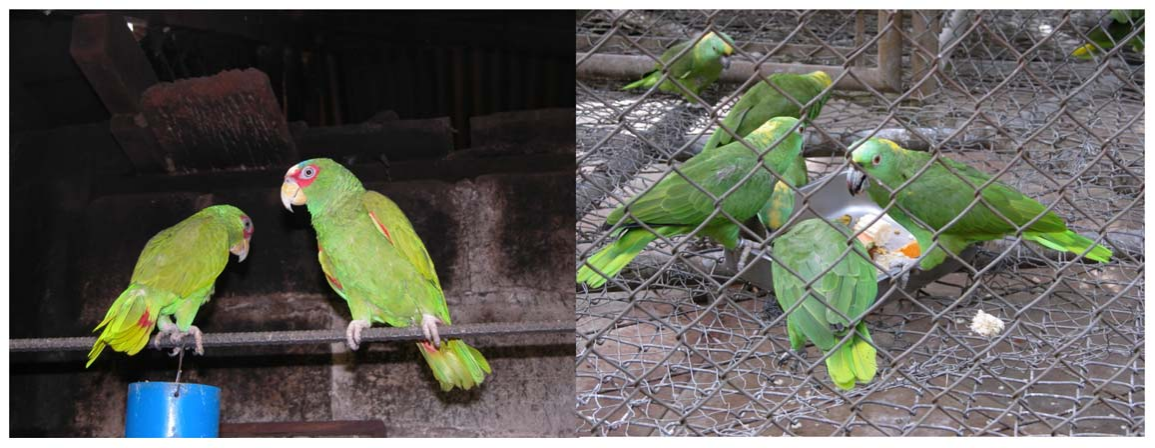
White-fronted (*Amazona albifrons*, left) and yellow-napped amazons (*Amazona auropalliata*, right) are often kept as household pets in Mexico and other Central American countries. About 8,000 and 1,000 individuals were illegally captured per year, respectively, in Mexico [18]. The yellow-napped amazon was listed by IUCN as Vulnerable in 2012 due to a rapid population decline (photos taken by J.L. Tella in Guatemala, 2005).

As measures of attractiveness of parrot species to pet owners, Pires and Clarke [12] used their body size and beauty (proportion of the bird that was brightly colored and the number of different plumage colors). These measures of attractiveness were supported by a previous independent study, showing that parrot species preferred by Europeans tended to be large and colorful [20]. However, and according to Pires [13], the ability to imitate human speech may be an important species-specific trait, making some parrot species more attractive as pets than others [21]. We predicted that amazon parrots (Genus *Amazona*, Fig. 1) and macaws (Genus *Ara*) would be preferred since they, along with the African grey parrot (*Psittacus erithacus*), are widely considered by parrot breeders and pet owners as those species most sought after for their ability to mimic human speech [22]. We thus grouped together species of Genus *Amazona* and *Ara* versus the rest of the species to obtain an additional measure of attractiveness (the ability to imitate human speech).

### Hypotheses Testing and Statistical Analyses

Instead of independently relating all CRAVED measures as ordinal-rank variables to the number of poached parrots through Kendall's Tau-b non-parametric correlations [12], we used multivariate modeling approaches to answer the following hierarchically-nested questions: 1) Were some species more attractive than others?; 2) Were attractive species more expensive?; 3) Were attractive species more heavily captured both for the legal and illegal pet market?; and 4) Does the over-capture of attractive species in the past affect their current conservation status?

We first performed a Categorical Principal Component Analysis (CATPCA) on beauty and body size (as provided by authors), and speaking ability of the species to obtain a single metric of species' attractiveness. A Generalized Linear Model (GLM) with normal distribution and identity link on CATPCA scores was used for testing the predicted differences in attractiveness between species (see above). We then related the attractiveness of species (CATPCA scores) to their prices, to the number of individuals illegally and legally traded, and finally to their current conservation status while controlling for variables reflecting their relative availability and accessibility.

Prices and numbers of parrots seized or traded followed a Poisson distribution. However, conditional variances were much larger than conditional means and thus data were better fitted to a negative binomial distribution (a particular case of the Poisson distribution), thus avoiding data overdispersion and inflation of parameter estimates in GLMs using the negative binomial distribution and the log link function. For assessing the relationships between numbers of seized/traded parrots and 1) their attractiveness and 2) their conservation status, we built sets of candidate models including all combinations of explanatory variables and their interactions. We then followed an information-theoretic model selection approach [23], computing the Akaike information criterion corrected for small sample sizes (AICc) and relative weight of evidence for each model (w_i_) as the probability of model _i_ being the best model for the observed data, given the set of candidate models. The most parsimonious model for each data set was selected based on a lower AICc and higher w_i_ (20). Models differing by < 2 units of AICc are considered as similarly explaining variability in the response variable. As a proxy of the variance explained, we calculated the percentage of deviance explained by the best supported models. All analyses were performed using SPSS v. 15.0. All data compiled as indicated above and used for analyses are shown in Appendix S1.

## Results

### Were Some Parrot Species More Attractive Than Others?

**Figure 2:**
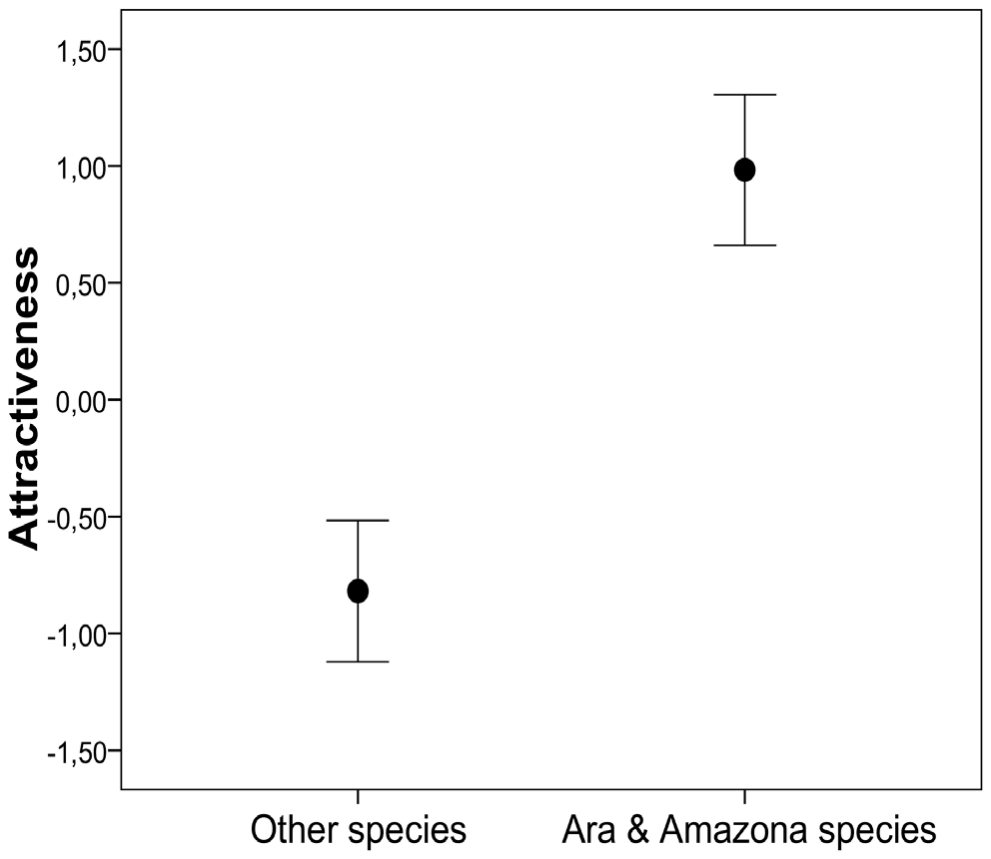
Attractiveness (mean and 95% CI) as the scores from a CATPCA performed on the beauty, body size and ability to talk of the 22 Mexican parrot species.

A CATPCA on the 22 species of Mexican parrots rendered a single dimension with an eigenvalue >1 (1.83), which correlated positively with the ability to talk (0.80), beauty (0.58) and body size (0.94) of the species, explaining 77.34% of the variance. The scores of this CATPCA can be interpreted as single descriptors of the attractiveness of parrot species to people, and were significantly larger for *Ara* and *Amazona* species (*mean*±*SE*: 0.98±0.14, *n* = 10) than for the rest of the species (−0.82±0.14, *n* = 12; GLM, Warld *χ^2^_1_* = 91.21, *P*<0.001, 84.4% of deviance explained). These scores did not overlap between the two groups of species (Fig. 2).

### Were Attractive Species More Expensive?

**Figure 3:**
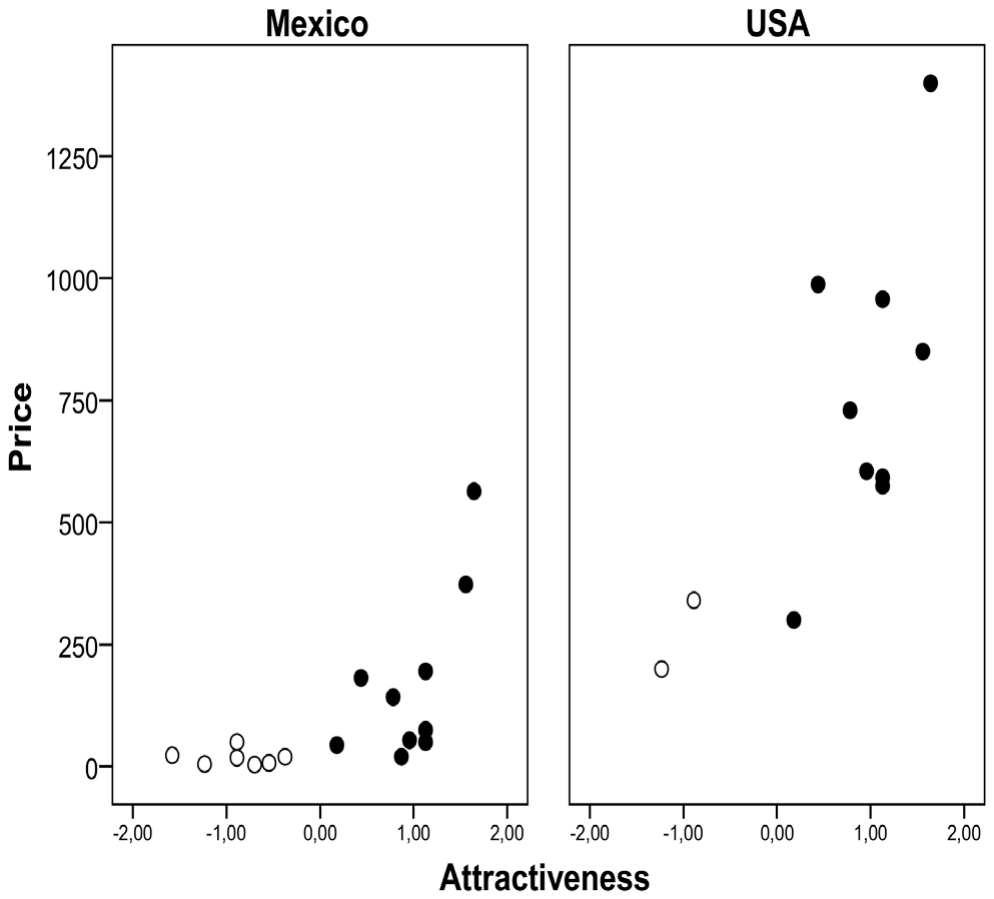
Average price (in US dollars) of Mexican parrots in Mexico and the USA in relation to their attractiveness. Attractiveness scores are those shown in Figure 2. Prices were recorded in 1999–2006. Black dots represent *Ara* and *Amazona* species, while empty dots represent other parrot species.

Prices were available for 17 species in Mexico and 11 species in the USA. When controlling for species identity in a GLM, prices were higher in the USA (estimated marginal mean, EMM: $ 382±40) than in Mexico ($ 58±5, Warld *χ^2^_1_* = 120.1, *P*<0.001) and in both cases correlated positively with attractiveness (estimate ± SE: 0.85±0.08, Warld *χ^2^_1_* = 104.4, *P*<0.001; 94.64% of deviance explained) (Fig. 3). A GLM controlling for price differences between countries (Warld *χ^2^_1_* = 41.88, *P*<0.001) showed that prices of *Ara* and *Amazona* species (EMM: $ 374±67) were on average six times higher than prices of other parrots ($ 62±14) (Warld *χ^2^_1_* = 36.54, *P*<0.001, 71.12% of deviance explained). Therefore, *Ara* and *Amazona* species were more attractive and valuable than the other parrots.

### Were Attractive Species Disproportionally Over-captured?

**Figure 4:**
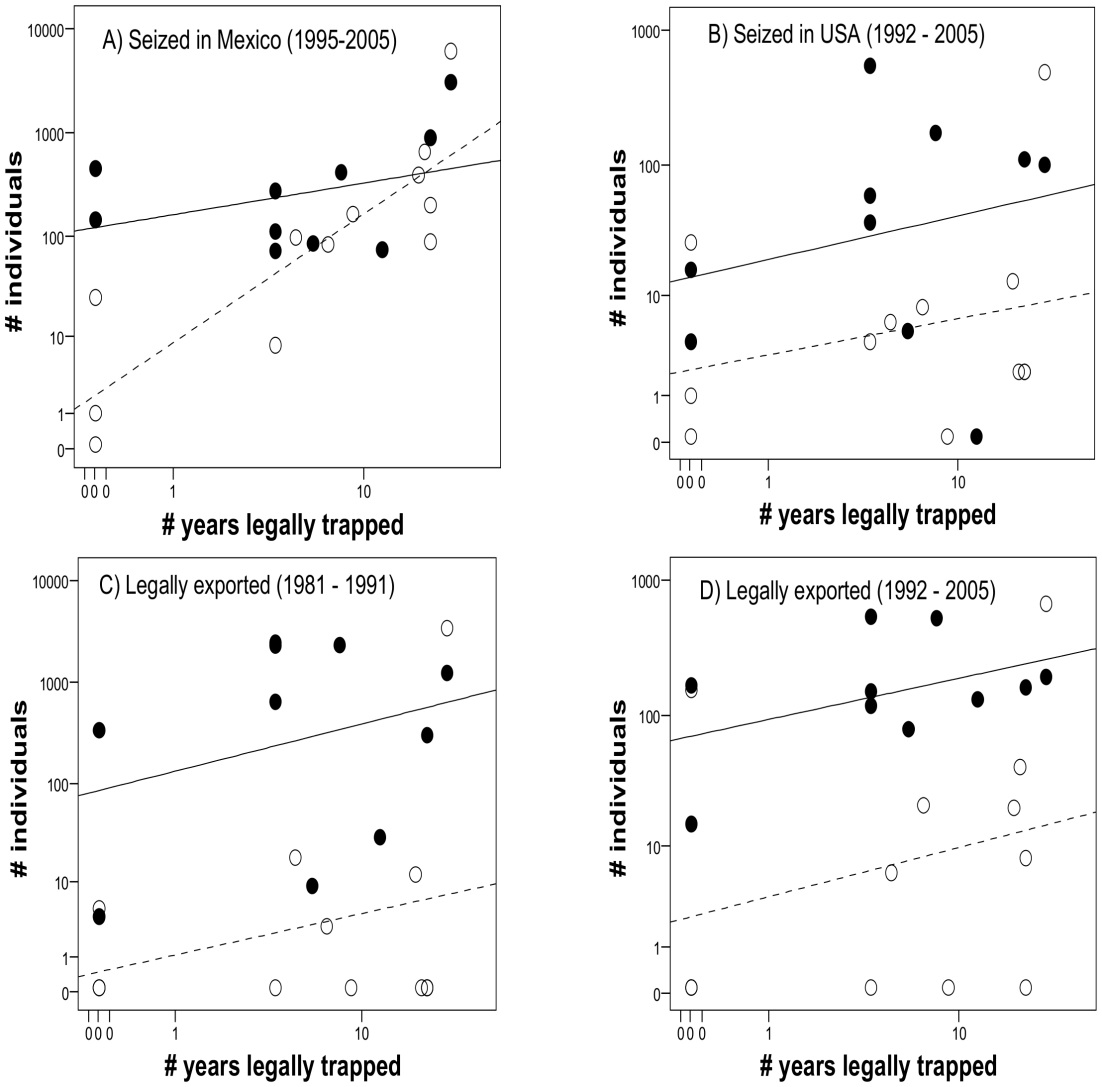
Number of Mexican parrots illegally (A and B) and legally (C and D) traded related to their attractiveness and the number of years the capture of each species was legally allowed in Mexico. Black dots represent the most attractive (*Ara* and *Amazona)* species, while empty dots represent other parrot species. Data are log-transformed since they fit a Poisson-like distribution. Regression lines are fitted for each group of species. Note that for panel D the best supported model related the number of traded parrots to their attractiveness and nest accessibility (Table 1), but the number of years legally trapped is shown for a better comparison with panels A–C.

The best supported GLM (Table 1) for explaining variability in the number of illegally caught parrots seized in Mexico between 1995 and 2005 (*n* = 13,375) shows that the most attractive species (*Ara* and *Amazona*) were more frequently poached when controlling for the positive effect of the number of years their capture was legally allowed, with a significant interaction between species attractiveness and number of years. This model explained 66.81% of the deviance and shows that attractive species were captured more than expected attending to their relative legal availability (Fig. 4A).

**Table 1:** GLM models obtained for explaining the number of Mexican parrots seized or legally exported attending to their attractiveness and three other species-specific variables.

MODELS	ATTRACT	YEARS	ATTRACT x YEARS	NEST	AICc	ΔAICc	w_i_	% dev
**Seized in Mexico (1995–2005)**								
ATTRACT + YEARS + (ATTRACT x YEARS)	16.15 ***	41.97 ***	6.69 **		288.44	0	0.8405	66.81
ATTRACT + YEARS					291.91	3.47	0.1483	
YEARS					297.13	8.69	0.0109	
OVERLAP + NEST					304.24	15.8	0.0003	
NEST					307.35	18.91	7E-05	
**Seized in USA (1992 – 2005)**								
ATTRACT + YEARS	7.98 **	3.80 *			220.88	0	0.7509	22.90
ATTRACT + NEST					224.02	3.14	0.1562	
OVERLAP					225.06	4.18	0.0929	
**Legally exported (1981 – 1991)**								
ATTRACT + YEARS	67.05 ***	18.27 ***			279.63	0	0.9539	34.93
ATTRACT + NEST					285.73	6.1	0.0452	
NEST + OVERLAP					293.48	13.85	0.0009	
**Legally exported (1992 – 2005)**								
ATTRACT + NEST	6.20 **			3.61 *	253.58	0	0.6356	14.90
ATTRACT + YEARS					255.84	2.26	0.2053	
NEST + OVERLAP					256.35	2.77	0.1591	

Wald *χ^2^* and statistical significance (*P*-values: * 0.05, ** 0.01, *** 0.001) of retained explanatory variables and the percentage of deviance explained (% dev) are only shown for the best-supported models, since all alternative models showed > 2 units of change in AICc. For simplicity, only candidate models with ΔAICc < 20 are shown. Explanatory variables are abbreviated as follows: ATTRACT (attractiveness), YEARS (number of years the capture of the species was legally allowed), NEST (accessibility of nests), and OVERLAP (overlap between the distribution of species and human populations in Mexico). Interactions between variables are denoted by “x”.

Poached parrots seized at the US border between 1992 and 2005 (*n* = 1,600) showed a similar pattern (Fig. 4B). The best supported GLM (Table 1, 22.90% of deviance explained) shows that the most attractive species were more frequently smuggled when controlling for the number of legal trapping years.

The same pattern arose for parrots legally exported from Mexico before (*n* = 13,051, explained deviance: 34.93%, Fig. 4C) and after 1992 (*n* = 2,993, explained deviance: 14,90%; Fig. 4D). In the latter case, however, the best-supported model (Table 1) shows more trade of the most attractive species when controlling for the accessibility of their nests (species with more accessible nests were more often exported) instead of for the number of trapping years. Nonetheless, the model including the number of legal trapping years was closely supported (ΔAICc = 2.26, Table 1).

### Were Currently Threatened Species Over-captured In The Past?

**Figure 5:**
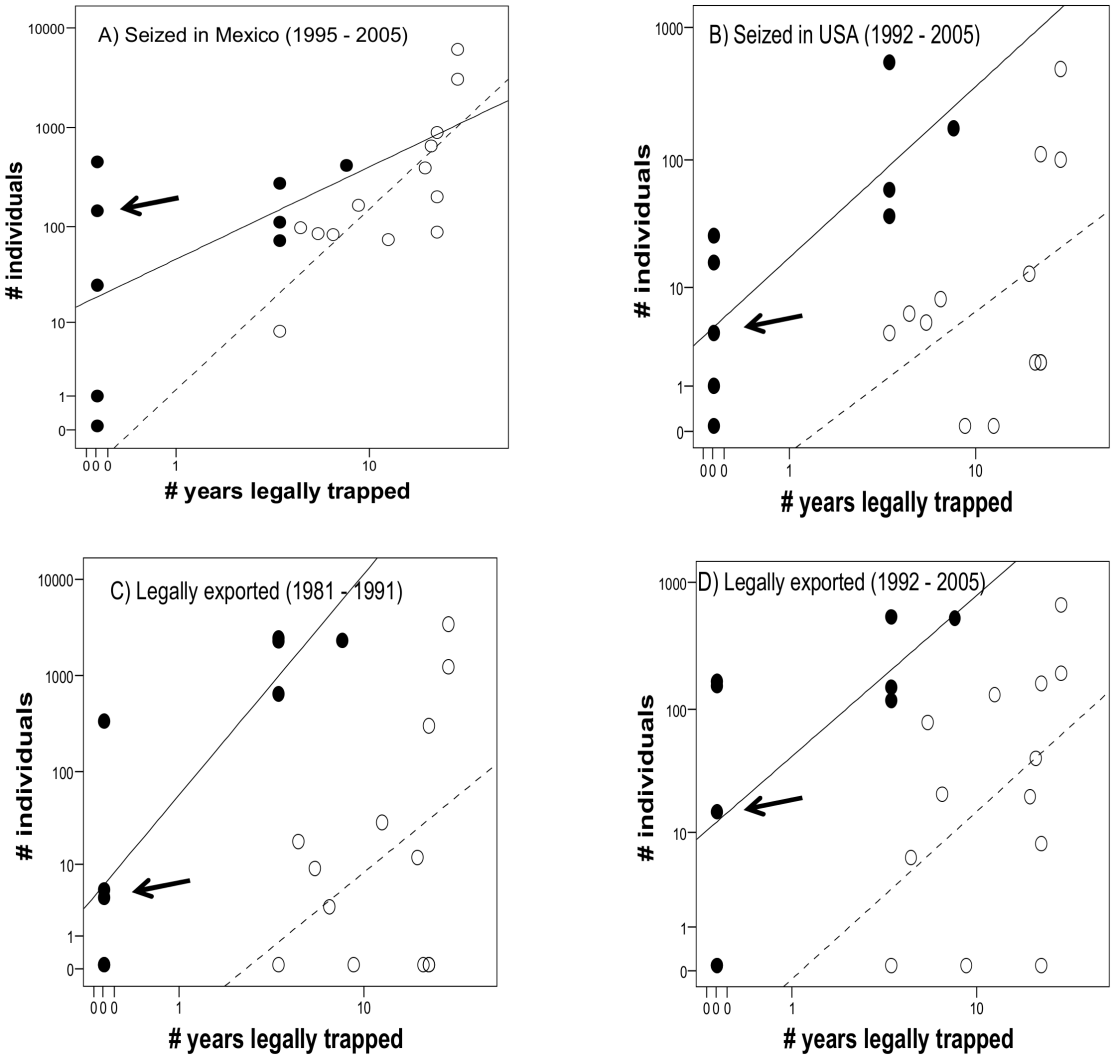
Number of Mexican parrots illegally (A and B) and legally (C and D) traded related to their current conservation status and the number of years the capture of each species was legally allowed in Mexico. Black dots represent threatened (Vulnerable and Endangered) species while empty dots represent non-threatened species according to IUCN (2013). Arrows indicate the scarlet macaw (*Ara macao*), a species that is not globally threatened but whose subspecies endemic to Central America is Endangered. Results excluding this species (not shown) are nearly identical. Data are log-transformed since they fit a Poisson-like distribution. Regression lines are fitted for each group of species.

Species threatened in 2013 (*n* = 9) were more often legally and illegally traded in the past than non-threatened species (*n* = 13) when controlling for surrogates of their relative legal availability and accessibility (Fig. 5). The best-supported models always included current threat status and number of years legally trapped, as well as the overlap between the distribution of parrots and humans for parrots seized in the USA between 1992 and 2005, and the interaction between threat status and overlap for parrots legally exported between 1981 and 1991 (Table 2). Models explained over 50% of the deviance, dropping to 23% in the case of parrots legally exported after 1992 (Table 2).

**Table 2:** GLM models obtained for explaining the number of Mexican parrots seized or legally exported in the past attending to their current conservation status and three other species-specific variables.

MODELS	THREAT	YEARS	THREAT x YEARS	OVERLAP	AICc	ΔAICc	W_i_	% dev
**Seized in Mexico (1995–2005)**								
THREAT + YEARS	13.18 ***	34.98 ***			293.64	0	0.99399057	55.37
NEST + OVERLAP					304.24	10.6	0.0049616	
NEST					307.35	13.71	0.00104784	
**Seized in USA (1992 – 2005)**								
THREAT + YEARS + OVERLAP	19.90 ***	11.96 ***		11.04***	197.7	0	0.97859031	53.9
THREAT + YEARS					205.39	7.69	0.02092853	
NEST + OVERLAP					212.94	15.24	0.00048004	
**Legally exported (1981 – 1991)**								
THREAT + YEARS + OVERLAP + (THREAT x YEARS)	4.34 *	28.24 ***	4.33 *	17.89 ***	261.59	0	0.95050301	51.14
THREAT + YEARS + (THREAT x YEARS)					268.2	6.61	0.03488271	
THREAT + YEARS					269.94	8.35	0.01461417	
**Legally exported (1992 – 2005)**								
THREAT + YEARS	9.32 **	9.45 **			249.99	0	0.96007467	23.24
NEST + OVERLAP					256.35	6.36	0.03992533	

Wald *χ^2^* and statistical significance (*P*-values: * 0.05, ** 0.01, *** 0.001) of retained explanatory variables and the percentage of deviance explained (% dev) are only shown for the best-supported models, since all alternative models showed > 2 units of change in AICc. For simplicity, only candidate models with ΔAICc < 20 are shown. Explanatory variables are abbreviated as follows: THREAT (currently threatened), YEARS (number of years the capture of the species was legally allowed), NEST (accessibility of nests), and OVERLAP (overlap between the distribution of species and human populations in Mexico). Interactions between variables are denoted by “x”.

## Discussion

Pires and Clarke [12] merited bridging the gap between conservation criminology and conservation biology by addressing a widespread issue (the illegal wildlife trade) from a criminology perspective, using parrots as the group of birds more severely affected by the legal and illegal trade [2]. A recent study on the human perception of beauty of parrots showed that zoos preferentially keep colorful and large-sized species which are preferred by the public [20]. These results suggested that parrot poaching may also be influenced by the attractiveness of particular species to humans [20], [21]. To our knowledge, Pires and Clarke [12] were the first to test whether species are poached according to their availability/accessibility in the wild (indicating an opportunistic crime) or whether some species are disproportionally caught because they are more enjoyable and valuable. While the poor knowledge on population sizes of tropical parrots [2] made difficult this approach, Pires and Clarke [12] obtained proxies of abundance and accessibility of species that correlated quite well with the estimated numbers of parrots poached in Mexico. Positive correlations with the accessibility of nests and the spatial overlap between parrot distributions and human populations suggested that the more accessible and removable species were more poached [12]. The stronger correlation between the number of parrots poached and the number of years each species was legally allowed to be trapped was interpreted as a positive relationship with their relative abundance in the wild [12]. Cantú *et al*. [18], however, pointed out that the annual allowance of permits to capture parrots was not based on species-specific population size studies. Although there is a general pattern sowing that the commonest species were allowed to be legally trapped more years, this pattern did not fit well for some species [18] and there is the possibility that authorities extended legal trapping of some economically important species regardless of their conservation status. Therefore, this variable should be interpreted as a measure of the legal availability of parrot species rather than as their relative abundance in the wild.

The above results moved Pires and Clarke [12] to conclude that parrot poaching in Mexico is a predominantly opportunistic crime, since the most widely available species and those captured most easily were taken in greater numbers, while the more expensive and rare species were taken in much smaller numbers. However, their univariate statistical approach together with the mixture of variables (price and conservation status were scored together) did not allow for testing the selective uptake of preferred species, while controlling for their availability and accessibility. Our multivariate and hierarchical hypotheses-based analyses of the same data and additional information on the legal and illegal parrot trade in Mexico shows however the selective uptake of particular species. Parrot species widely differed in attractiveness as reflected by their combined measures of body size, coloration, and ability to talk. As expected, given the human preference for large-bodied and colorful parrot species [20] and the ability to imitate human speech in amazons and macaws [22], the attractiveness scores of the species strongly correlated with their prices both in the Mexican and US markets. Finally, the most attractive species were more captured than expected when controlling for measures of their relative legal availability (number of years of trapping) and accessibility. These results strongly support the selective poaching of the most enjoyable and valuable species rather than just an opportunistic poaching of the commonest ones. Notably, similar patterns were found for parrots poached for the domestic Mexican market, for those smuggled to the USA, and for those legally exported before or after 1992, when the USA ban led parrot exports to be restricted to other countries (mostly Europeans, [8]). Therefore, preferences for the same attractive species seem to hold true among the culturally and economically different societies of Mexico, North America and Europe. As it has been shown for boid snakes, a cross-cultural agreement in perception of animal attractiveness [24] seems to cause the selective capture of parrot species for the worldwide demand of pets and cage birds.

Whether parrot poaching is selective or just an opportunistic activity is not a trivial question. The opportunistic uptake of parrots would rest conservation concern, since it would mostly affect to the more available and less threatened species as suggested by results obtained by Pires and Clarke [12]. On the other hand, the selective poaching of attractive parrot species is an intuitive-appealing hypothesis, as Munn [21] suggested in his review of parrot conservation and trade: “The species endangered by trade are invariably colorful, large, or good talkers. In fact, the reason that the large, colorful, talkative species of wild parrots are in trouble is not because of shortage of habitat but because so many humans like to keep them as pets”. There were, however, few attempts at testing this hypothesis and it received little empirical support. Wright *et al*. [7] did not find a correlation between the level of nest poaching experienced by 16 species of Neotropical parrots and their retail prices in US, although poaching rates were higher in species with retail prices above $ 500. These results came from field studies conducted in 14 countries, which could mask a positive trend due to differences among countries in poaching pressure and the development of international trade [2]. Our results, obtained from a single country, show a strong correlation between species attractiveness and their prices (ca. 95% of deviance explained). Moreover, there is evidence that several parrot species declined due to wildlife trade, and the capture and smuggling of the last Spix's macaws caused the definitive extinction of the species in the wild in October 2000 [5]. To our knowledge, however, a link between the attractiveness of parrot species and their extinction risk was not previously demonstrated through comparative analyses. Although analyses using proxies of the availability and accessibility of parrot species, in the absence of wild population estimates, must be taken with caution, our results show that attractive species tend to be more threatened with extinction. We could infer causality from our analyses, since most currently threatened parrots were over-captured considering the number of years they were legally allowed to be trapped decades before they became threatened (1981–1991; Fig. 5C). In fact, six of the studied species (*Ara militaris, Amazona oratrix, Amazona viridigenalis, Aratinga brevipes, Rhynchopsitta pachyrryncha, Rhynchopsitta terresi*) were first listed as globally threatened in 1994, one (*Amazona finschi*) in 2006 and one (*Amazona auropalliata*) in 2012 (www.iucnredlist.org), while *Ara macao* has officially been considered endangered in Mexico since 1994 [18]. These six amazons and macaw species show positive attractiveness scores (ranging from 0.44 to 1.65), and excessive captures for the domestic and international trade were reasons for listing these species as threatened (www.iucnredlist.org, [18]). Therefore, our results empirically support previous IUCN decisions. However, *A. brevipes, R. pachyrryncha,* and *R. terresi* show low attractiveness (scores ranging from −0.29 to −0.72), and accordingly were rarely poached or traded (see Appendix S1); in these cases, their listing as a threatened species was due to habitat degradation in their naturally restricted range distributions (www.iucnredlist.org). The increasing rarity of these species might make them even more attractive to private collectors, thus increasing their risk of extinction through an anthropogenic Allee effect [17], [25]. Nonetheless, preferred species (macaws and Amazon parrots) are now widely bred in captivity and the supply is greater than demand in the USA and Europe, where their prices are dropping ([18], J.L. Tella, unpubl. data). Although some of these species were already bred in captivity by ancient Mexican cultures 600–800 yr ago [26], they are now rarely bred in Mexico making their prices poorly competitive [18]. Therefore, a greater pressure is expected from the domestic illegal trade than from international smuggling.

Conservation biologists have been justifiably concerned for decades regarding the international trade on wild parrots [2]–[8]. While the conservation value of wildlife trade bans led to a heated debate on their potential to boost illegal international trade [4], [27], [28], international trade in parrots has in fact been drastically reduced since US and European bans were enforced ([8] www.cites.org). However, the domestic demand of parrots seems to cause high poaching rates unrelated to international trade [18], [29], with the conservation problem often overlooked. Cantú *et al.* [18] estimated that 65,000–78,500 parrots are poached annually in Mexico, 86–96% being sold domestically. While pre-Colombian Mexican cultures captured and kept parrots in captivity [13], [26], the economic upsurge in the last decades may have led to an unsustainable increase in this activity. In fact, recent studies reported a sharp reduction of Mexican parrot distributions despite a prevalence of suitable habitats [30]. The same figure may apply to other countries such as Brazil, Bolivia, Venezuela and Peru where large parrot poaching activities to satisfy the domestic demand have recently been reported [29], [31]–[35]. Urgent research and conservation work is thus required to save parrot populations from decimation due to the domestic demand of pets and the lack of law enforcement of trade in those countries. On the one hand, poaching levels should be related to the relative abundance of parrot species in the wild, using available census methodologies (e.g., [29], [36]–[38], to ascertain their actual impact on threatened species. On the other hand, awareness campaigns must be addressed to local populations to halt the uptake of declining species. This will require much effort, since keeping parrots as pets is a long standing tradition [13] and the preference for the most attractive species seems to be widespread among cultures.

## Supporting Information

Appendix S1 **Raw data used for statistical analyses and their sources.** Number of poached parrots seized in Mexico and USA [18]; number of parrots legally traded before and after 1992 (www.cites.org); number of years the capture of the species was legally allowed, overlap between the distribution of species and human populations in Mexico, and accessibility of nests [12]; prices (in $) in Mexico and USA [18]; conservation status in 2013 ([19], www.iucnredlist.org); beauty scores and size (in cm) of species [12], and their ability to mimic human speech [22].
(DOC)
