# Supplementary material for: Illegal and Legal Parrot Trade Shows a Long-Term, Cross-Cultural Preference for the Most Attractive Species Increasing Their Risk of Extinction
Source: PLoS One. 2014 Sep 16;9(9):e107546. doi: 10.1371/journal.pone.0107546 (PMC4166463; doi:10.1371/journal.pone.0107546)
Supplement: Appendix S1 — Raw data used for statistical analyses and their sources. Number of poached parrots seized in Mexico and USA [18]; number of parrots legally traded before and after 1992 (www.cites.org); number of years the capture of the species was legally allowed, overlap between the distribution of species and human populations in Mexico, and accessibility of nests [12]; prices (in $) in Mexico and USA [18]; conservation status in 2013 ([19], www.iucnredlist.org); beauty scores and size (in cm) of species [12], and their ability to mimic human speech [22]. (DOC) [file pone.0107546.s001.doc]

| **Species** | **Seized in Mexico (1995 - 2005)** | **Seized in**  **USA (1992 - 2005)** | **Legally traded (1981 - 1991)** | **Legally traded (1992 - 2005)** | **# of years trapped** | **Overlap** | **Nest accesibility** | **Price**  **In**  **Mexico** | **Price in USA** | **Threatened** | **Beauty** | **Body size** | **Good talker** |
| --- | --- | --- | --- | --- | --- | --- | --- | --- | --- | --- | --- | --- | --- |
| *Amazona albifrons* (white-fronted amazon) | 3062 | 100 | 1233 | 193 | 23 | 15 | 1 | 44 | 300 | No | 1 | 25 | Yes |
| *Amazona auropalliata* (yellow-naped amazon) | 72 | 37 | 645 | 150 | 4 | 18 | 1 | 182 | 988 | Yes | 1 | 32 | Yes |
| *Amazona autumnalis* (red-lored amazon) | 891 | 110 | 300 | 161 | 19 | 21 | 0 | 75 | 592 | No | 2 | 33 | Yes |
| *Amazona farinosa* (mealy amazon) | 85 | 5 | 9 | 79 | 6 | 13 | 0 | 142 | 730 | No | 1 | 39 | Yes |
| *Amazona finschi* (lilac-crowned amazon) | 415 | 173 | 2317 | 525 | 8 | 9 | 1 | 50 | 575 | Yes | 2 | 33 | Yes |
| *Amazona oratrix* (yellow-headed amazon) | 274 | 546 | 2286 | 538 | 4 | 22 | 0 | 195 | 957 | Yes | 2 | 33 | Yes |
| *Amazona viridigenalis* (green-cheeked amazon) | 111 | 59 | 2442 | 118 | 4 | 14 | 0 | 54 | 605 | Yes | 2 | 30 | Yes |
| *Amazona xantholora* (yellow-lored amazon) | 74 | 0 | 29 | 131 | 12 | 10 | 0 | 20,4 |  | No | 2 | 26 | Yes |
| *Ara macao* (scarlet macaw) | 144 | 4 | 4 | 15 | 0 | 2 | 0 | 564 | 1400 | Yes | 2 | 88 | Yes |
| *Ara militaris* (military macaw) | 451 | 16 | 336 | 167 | 0 | 16 | 0 | 373 | 850 | Yes | 2 | 85 | Yes |
| *Aratinga brevipes* (Socorro conure) | 0 | 0 | 0 | 0 | 0 | 1 | 0 |  |  | Yes | 1 | 32 | No |
| *Aratinga canicularis* (orange-fronted conure) | 6085 | 486 | 3412 | 666 | 23 | 11 | 1 | 18 |  | No | 1 | 24 | No |
| *Aratinga holochlora* (green conure) | 391 | 13 | 12 | 20 | 17 | 6 | 0 | 20 |  | No | 1 | 35 | No |
| *Aratinga nana* (Aztec conure) | 654 | 2 | 0 | 41 | 18 | 19 | 1 | 23 |  | No | 0 | 23 | No |
| *Aratinga strenua* (Pacific conure) | 164 | 0 | 0 | 0 | 9 | 7 | 1 | 4 |  | No | 2 | 17 | No |
| *Bolborhynchus lineola* (barred parakeet) | 88 | 2 | 0 | 8 | 19 | 17 | 0 |  |  | No | 0 | 17 | No |
| *Brotogeris jugularis* (orange-chinned parakeet) | 200 | 2 | 0 | 0 | 19 | 5 | 1 | 7 |  | No | 1 | 32 | No |
| *Forpus cyanopygius* (Mexican parrotlet) | 97 | 6 | 18 | 6 | 5 | 8 | 0 | 5 | 200 | No | 1 | 13 | No |
| *Pionopsitta haematotis* (brown-hooded parrot) | 8 | 4 | 0 | 0 | 4 | 12 | 0 |  |  | No | 2 | 22 | No |
| *Pionus senilis* (white-crowned parrot) | 83 | 8 | 3 | 21 | 7 | 20 | 0 | 50 | 340 | No | 1 | 24 | No |
| *Rhynchopsitta pachyrhyncha* (thick-billed parrot) | 25 | 26 | 5 | 154 | 0 | 4 | 0 |  |  | Yes | 1 | 38 | No |
| *Rhynchopsitta terresi* (maroon-fronted parrot) | 1 | 1 | 0 | 0 | 0 | 3 | 0 |  |  | Yes | 0 | 43 | No |
|  |  |  |  |  |  |  |  |  |  |  |  |  |  |
